# Deep learning-based image annotation for leukocyte segmentation and classification of blood cell morphology

**DOI:** 10.1186/s12880-024-01254-z

**Published:** 2024-04-08

**Authors:** Vatsala Anand, Sheifali Gupta, Deepika Koundal, Wael Y. Alghamdi, Bayan M. Alsharbi

**Affiliations:** 1 Chitkara University Institute of Engineering and Technology, Chitkara University, Rajpura, Punjab India; 2 School of Computer Science, University of Petroleum & Energy Studies, Dehradun, India; 3 Ho Chi Minh City Open University, Ho Chi Minh City, Vietnam; 4 Department of Computer Science, College of Computers and Information Technology, Taif University, P. O. Box 11099, 21944 Taif, Saudi Arabia; 5 Department of Information Technology, College of Computers and Information Technology, Taif University, P. O. Box 11099, 21944 Taif, Saudi Arabia

**Keywords:** Leukocytes, Diseases, Leukemia, Deep learning, White blood cells, Segmentation

## Abstract

The research focuses on the segmentation and classification of leukocytes, a crucial task in medical image analysis for diagnosing various diseases. The leukocyte dataset comprises four classes of images such as monocytes, lymphocytes, eosinophils, and neutrophils. Leukocyte segmentation is achieved through image processing techniques, including background subtraction, noise removal, and contouring. To get isolated leukocytes, background mask creation, Erythrocytes mask creation, and Leukocytes mask creation are performed on the blood cell images. Isolated leukocytes are then subjected to data augmentation including brightness and contrast adjustment, flipping, and random shearing, to improve the generalizability of the CNN model. A deep Convolutional Neural Network (CNN) model is employed on augmented dataset for effective feature extraction and classification. The deep CNN model consists of four convolutional blocks having eleven convolutional layers, eight batch normalization layers, eight Rectified Linear Unit (ReLU) layers, and four dropout layers to capture increasingly complex patterns. For this research, a publicly available dataset from Kaggle consisting of a total of 12,444 images of four types of leukocytes was used to conduct the experiments. Results showcase the robustness of the proposed framework, achieving impressive performance metrics with an accuracy of 97.98% and precision of 97.97%. These outcomes affirm the efficacy of the devised segmentation and classification approach in accurately identifying and categorizing leukocytes. The combination of advanced CNN architecture and meticulous pre-processing steps establishes a foundation for future developments in the field of medical image analysis.

## Introduction

Within the domain of restorative imaging and diagnostics, the examination of blood cell morphology stands as a foundation for understanding and diagnosing different illnesses. Among the horde of blood cells, leukocytes, show a significant part within the resistant system's defense against toxicities and infections. The complex examination of leukocyte morphology, in any case, postures a noteworthy challenge to conventional demonstrative strategies [1].

Leukocyte division and classification represent urgent components within the space of medical image investigation, especially within the setting of hematology. As basic components of the resistant framework, leukocytes, or white blood cells, play an essential part in guarding the body against contaminations and maladies. The precise recognizable proof and classification of leukocytes contribute essentially to demonstrative forms, helping within the early discovery of different wellbeing conditions [2, 3].

The method of leukocyte division includes the confinement and depiction of person white blood cells inside complex microscopic images. This errand is inalienably challenging due to the different run of cell shapes, sizes, and the potential cover of cells in thick regions of the images. Over a long time, analysts have investigated numerous approaches to address these challenges [4–6].

A noteworthy jump within the exactness of leukocyte division has been seen with the coming of profound learning, particularly CNNs. The conventional ways of checking WBCs take a long time and require the help of medical experts. In this way, there's a need for a framework that can consequently analyze white blood cells through the forms of division, and classification [7, 8]. Division of leukocytes from blood cell images is the objective that points to extricate the vital highlights for consequent preparing. Recognizing the potential for development, this investigate sets out on a comprehensive investigation of the division and classification of leukocytes, leveraging the capabilities of profound learning inside the space of restorative image examination.

Traditional diagnostic methods for classifying leukocytes in blood cell images face several challenges. Human interpretation of blood cell images can vary based on the observer's experience and training, leading to inconsistencies in diagnosis. Manual classification of leukocytes in blood cell images is a labor-intensive process that requires trained personnel, making it time-consuming and costly. Manual classification is not easily scalable to large datasets, limiting its utility in processing a high volume of images efficiently. Moreover, blood cell images may contain artifacts or overlapping cells, making it challenging for traditional methods to accurately classify leukocytes.

Deep learning offers a promising solution to the challenges faced by traditional methods in classifying leukocytes in blood cell images. By leveraging neural networks, deep learning models can automatically learn relevant features from large datasets, reducing the reliance on human interpretation and the need for manual feature extraction [9].

The proposed research focuses on the image processing based segmentation and deep learning based classification of leukocytes, a crucial task in medical image analysis for diagnosing various diseases. The major offerings of the research include:

1. Leukocyte segmentation is achieved through image processing techniques, including background subtraction, noise removal, and contouring. For the leukocyte segmentation from blood sample images, background mask, erythrocyte mask and leukocyte mask are generated using morphological operations.
2. Bounding boxes are created around the segmented leukocyte region and are further cropped without taking background region and erythrocyte region in consideration. Isolated leukocytes are then subjected to data augmentation, including brightness and contrast adjustment, flipping, and random shearing, to improve the generalizability of the CNN model.
3. A deep convolution neural network based model having four convolutional blocks consisting of eleven convolutional layers, eight batch normalization layers, eight Rectified Linear Unit (ReLU) layers, and four dropout layers has been proposed to capture complex patterns for classification of leukocytes into its four different classes such as monocytes, lymphocytes, eosinophils, and neutrophils.

Rest of the paper is structured as: literature review is discussed in section "Literature Review", followed by proposed work in section "Proposed Work", section "Results and discussion" shows result and discussion, section "Conclusion and future work" displays conclusion and future work.

## Literature review

To adopt a novel way for automatically segmenting WBCs, a significant amount of study is taking place. In this section, brief descriptions of various WBCs algorithms are provided. Leukocyte segmentation is a time-consuming task because of the large range of cell shapes and imaging conditions. Leukocytes can be studied using several automated technologies, as indicated by the literature. Researchers are still trying to develop a system that can automatically segment leukocytes with higher accuracy in the shortest amount of time [10]. To separate the nucleus from the cytoplasm in blood smear images [11], used the stepwise averaging method using interval-valued fuzzy sets. By employing random forest classification, more than 95% accuracy was attained in the separation of the nucleus and the cytoplasm by authors [12, 13]. Leukemia diagnosis is predicted and diagnosed using classification algorithms based on leukocyte segmentation obtained from two separate blood smears utilizing the CMYK color space by [14]. Classification accuracy of 86.67% was achieved using support vector machines. The Bayes classifier-based method produced an overall accuracy of 80.88%. Author [15] examined the application of CNN classifiers, for identification and compare lymphocyte image cells. SVM and deep learning are used to classify abnormal blood cell images by [16]. In 2020, Zhana et al. [17] presented a new technique based on the thresholding segmentation technique for the segmentation of leukocytes from blood cell images. Shahin et al. [18] provided a complete end-to-end system for CNN to recognize the various WBCs classes. Two transfer learning techniques incorporate this mostly for the identification of WBCs. Mishra S et al. [19] developed an excellent approach for distinguishing normal WBCs. Linear Discriminant Analysis (LDA) was used to identify the textural characteristics and reduce the dimensionality of the dataset. Segmentation of leukocytes with higher accuracy in the shortest amount of time is necessary. Therefore, a precise model is required for the segmentation of leukocytes so that manual counting of leukocyte can be replaced with an intellectual mechanism. Here, in this study, an innovative deep learning-based model with advanced accurateness is proposed for cell segmentation, feature extraction, and classification of leukocyte that can replace traditional ways of counting leukocyte.

Although literature review methods can be used to generate good classification engines, but they still have some drawbacks. Traditional machine learning methods [10, 11, 14, 19] need to extract features manually. The acquisition of features mainly depends on the designer’s prior knowledge. This feature extraction method is difficult to make full use of the information contained in the image, and will increase the designer’s workload. The deep learning algorithm effectively solves this problem. It can automatically learn the effective features of the image. Deep learning algorithms such as deep residual network also have good performance in image classification tasks. However, these neural network classification algorithms cannot fully utilize some features of the image that have a long-term dependency relationship with image labels, and thus these classification methods cannot classify cell images like people with memory. Moreover, it is extremely hard to obtain a sufficient number of annotated and labelled images that can be used to train deep models in a given biomedical domain [17, 18]. There may be slight differences between two given images from a biomedical or medical area, and this could mean that the two images may indicate two different types of diseases. Here, an image processing based technique is introduced for segmentation and fuse it with a convolutional neural network to perform the task of blood cell image classification.

## Proposed work

**Fig. 1:**
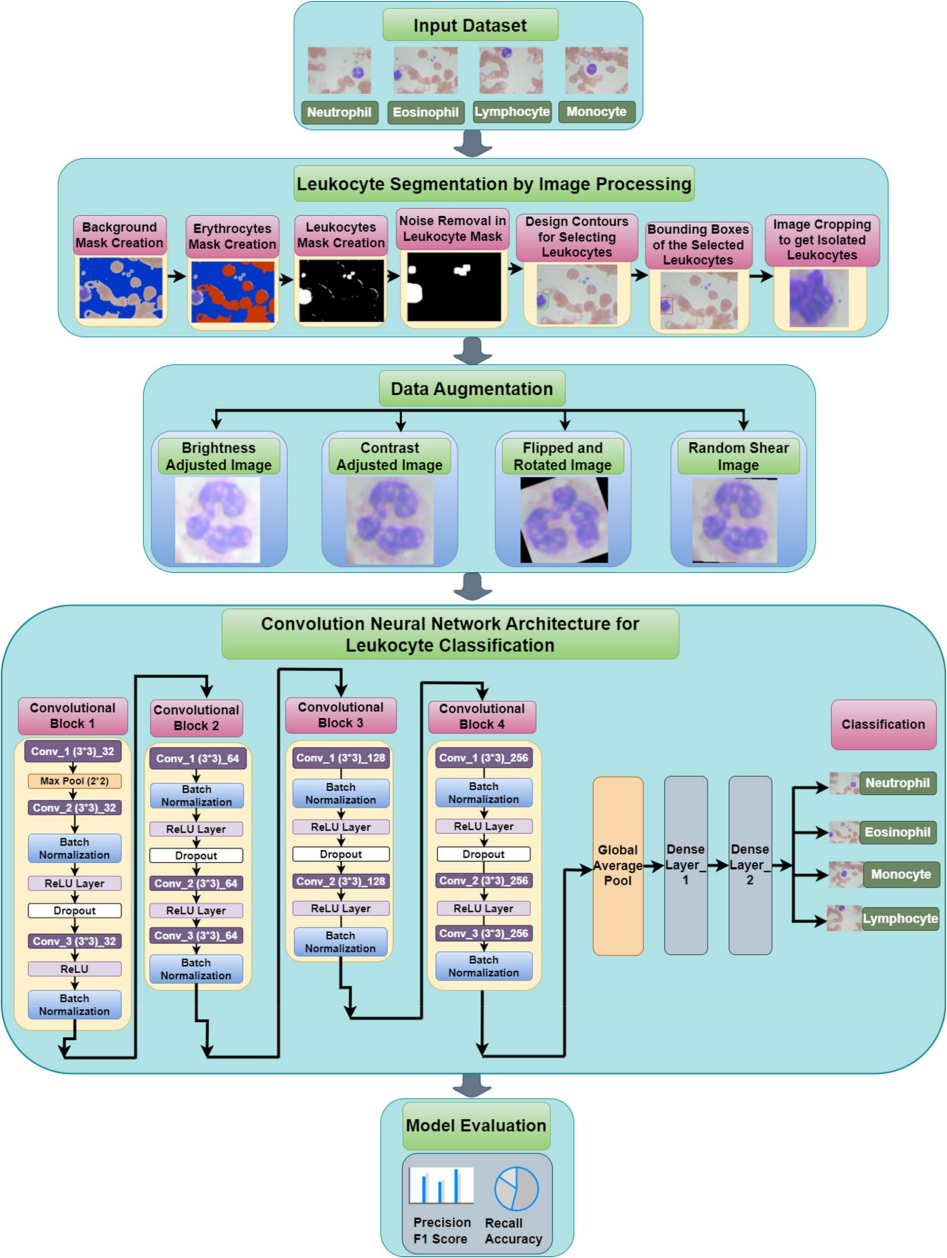
Pipeline of the Proposed Work

In this pipeline of the proposed work, leukocyte isolation by image segmentation is performed using various steps that are discussed in the following sections. Figure 1 displays the pipeline of the proposed work. The task of automatically classifying leukocytes in blood images is challenging due to the variability in their appearance. The figure breaks down leukocyte classification into several stages. First, the input dataset is preprocessed. The preprocessing workflow consists of three pivotal stages: the creation of Background Masks, Erythrocytes Masks, and Leukocytes Masks. Leukocyte segmentation is accomplished using image processing methods, encompassing procedures such as background subtraction, noise elimination, and contouring. Leukocytes undergo data augmentation techniques, which involve adjustments in brightness and contrast, flipping, and random shearing. These augmentation processes aim to enhance the overall adaptability and robustness of the CNN model.

Next, the preprocessed data is fed into the convolutional neural network. CNNs are a type of artificial neural network that are well-suited for image recognition tasks. The CNN in the figure consists of four convolutional blocks, each containing convolutional layers, a max pooling layer, and a ReLU layer. Convolutional layers extract features from the data, max pooling layers reduce the dimensionality of the data, and ReLU layers introduce non-linearity. The stride value for convolution and pooling layers is zero and no padding is applied in these layers. After each convolutional block, there is a batch normalization layer, which helps to improve the training speed and stability of the network.

Following the convolutional blocks, there are several fully connected layers. These layers take the output from the convolutional blocks and map it to the final class labels (neutrophil, eosinophil, lymphocyte, monocyte). Finally, the model is evaluated using metrics such as precision, recall, F1 score, and accuracy.

Overall, the convolutional neural network architecture in the figure provides a comprehensive approach to classifying leukocytes in blood images. By segmenting the leukocytes, preprocessing the data, and using a CNN with appropriate layers, the model can achieve high accuracy in classifying the different types of leukocytes. The proposed model is analysed using the Google Colab platform with Python.

### Input dataset

**Fig. 2:**
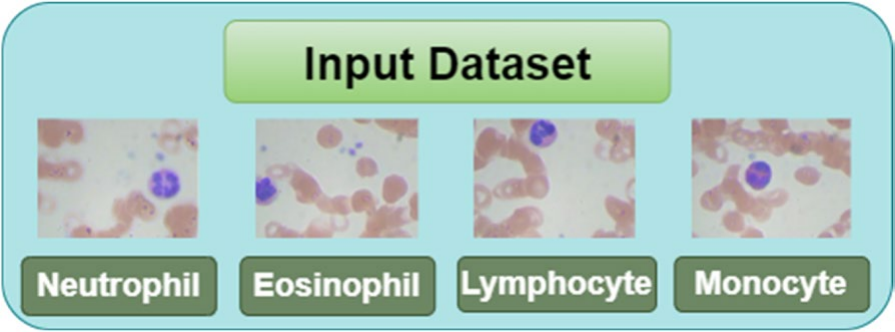
Samples of blood cell images

To validate the proposed model, blood samples have been collected from an online source. Blood sample images are collected from a publicly available dataset from Kaggle Mooney et al. [20]. The dataset contains four leukocyte types named Neutrophil (NE), Eosinophil (EO), Lymphocyte (LM), and Monocyte (MN) as shown in the Fig. 2. It consists of a total of 12,444 blood sample images out of which 3144 images belong to EO class, 3139 to LM, 3132 to MN and 3171 to NE class. The splitting ratio of 80:20 is used for training and testing of the model according to which the total count of training images is 9955 and the count of testing images is 2489. The size of the input image is 256 * 192. Figure 2 shows the blood samples obtained from the Kaggle dataset.

### Leukocyte isolation by image segmentation

**Fig. 3:**
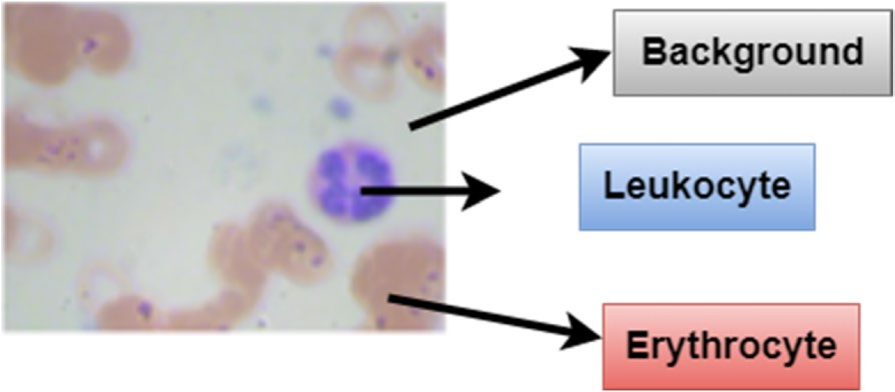
Parts of blood cell image

**Fig. 4:**
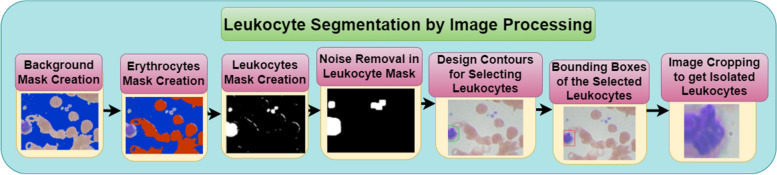
Leukocyte Segmentation by Image Processing

A blood cell image consists of three parts as shown in Fig. 3. First, the image undergoes background subtraction to remove erythrocytes and other non-leukocyte elements. Then, a noise removal step cleans up the image. Next, the leukocytes are segmented from the background using a mask creation process. Finally, the design corners are removed, leaving behind the isolated leukocytes. Here in the blood cell image, gray part is the background whereas, the leukocyte is shown in blue color and the dark brown part is the erythrocyte. For the segmentation of images and for isolating leukocytes, it is necessary to know the position of leukocytes in the training samples. For the detection of leukocytes, the processing is done by creating a mask for the background detection. After that, a mask is created for the detection of erythrocytes. Simple masks are created for filtering out the background area to extract leukocytes. Figure 4 shows the leukocyte isolation by image segmentation.

#### Background mask creation

In blood cell images, the background area does not contain erythrocytes as well as leukocytes and it is gray in color. The gray pixel in the image consists of the red, blue, and green components in the majority. For creating background mask, threshold (threshold_1) is calculated using Otsu thresholding method. So, the background mask is created using the following Eq. (1)

1 $${\text{Background}}\_\mathrm{mask }= ({\text{img}}[:, :, 0] >\mathrm{ threshold}\_1) \& ({\text{img}}[:, :, 1] >\mathrm{ threshold}\_1) \& ({\text{img}}[:, :, 2] >\mathrm{ threshold}\_1)$$

**Fig. 5:**
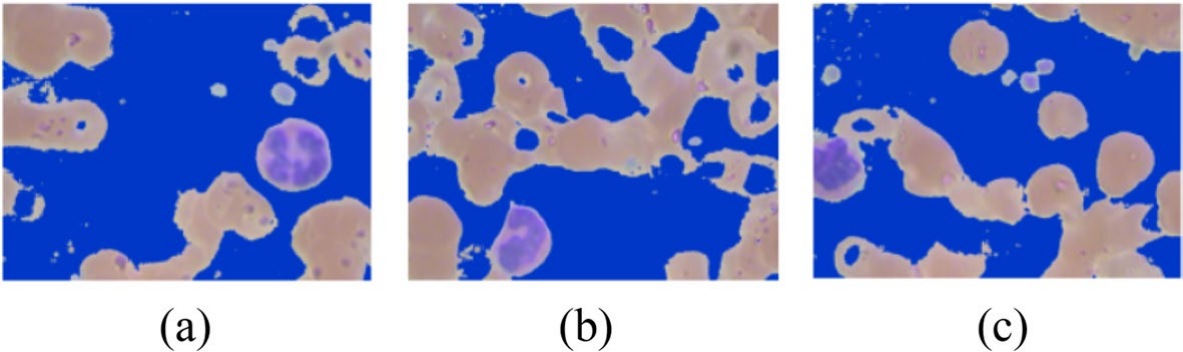
Leukocyte images with background region highlighted with blue color

In this equation, red component i.e. ${\text{img}}[:, :, 0]$, green component i.e. ${\text{img}}[:, :, 1]$ and blue component i.e. ${\text{img}}[:, :, 2$ all are having pixel value greater than threshold_1 whose value comes out to be 182 here. All background pixels satisfying the above equation are highlighted in blue color in Fig. 5.

#### Erythrocytes mask creation

In blood cell image shown in Fig. 3 erythrocyte is shown in light brown color. So, to extract the erythrocyte area from the blood cell images, the erythrocyte mask is created with a red component where the red pixel value is greater than the blue pixel value or the blue pixel value should be less than threshold value which comes out to be 150 calculated using Otsu thresholding method.

This function will detect the erythrocytes. So, the erythrocyte mask is created using the following Eq. (2)

2 $${\text{Erythrocyte}}\_\mathrm{mask }= ({\text{img}}[:, :, 2] <\mathrm{ threshold}\_2) | ({\text{img}}[:, :, 0] >\mathrm{ img}[:, :, 2])$$

**Fig. 6:**
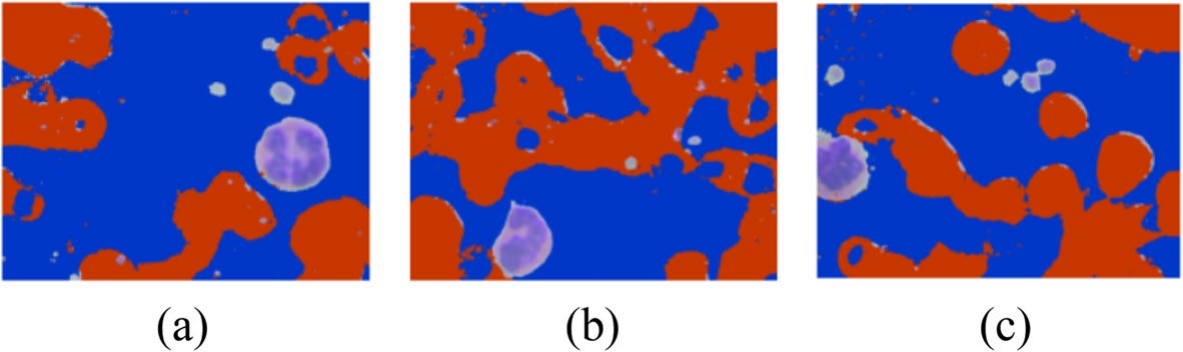
Leukocyte images with Erythrocytes region highlighted with red color

In this equation, blue component i.e. ${\text{img}}[:, :, 2]$ will always be less than red component i.e. ${\text{img}}[:, :, 2]$. Here, blue component i.e. ${\text{img}}[:, :, 2]$ is less than 150 or red component i.e ${\text{img}}[:, :, 2]$ is greater than blue component i.e. ${\text{img}}[:, :, 2]$. All erythrocyte pixels satisfying the above equation are highlighted in red color in Fig. 6.

#### Leukocytes mask creation

The leukocyte mask is created that does not include a background mask and erythrocyte mask. So, the leukocyte mask is created using the following Eq. (3)

3 $${\text{Leukocyte}}\_\mathrm{mask }= \sim {\text{is}}\_{\text{background}}({\text{img}}) \& \sim {\text{is}}\_{\text{erytrocyte}}({\text{img}})$$

**Fig. 7:**
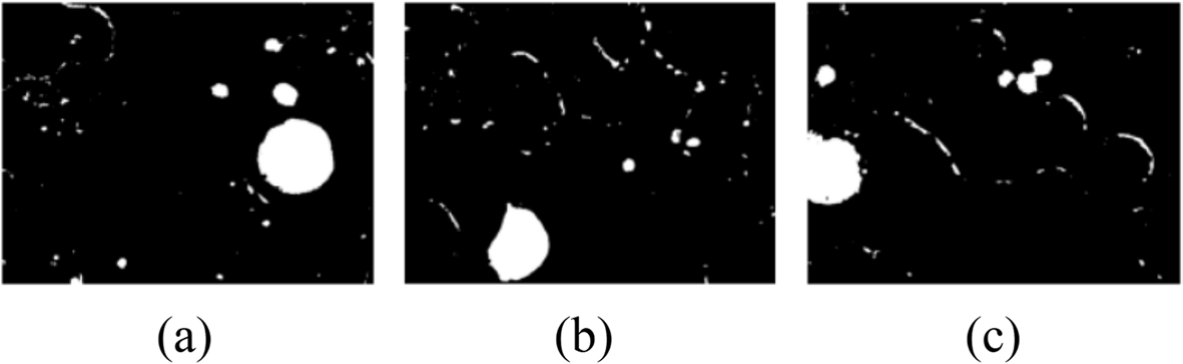
Leukocyte mask

Figure 7 shows the image samples with leukocyte masks. From Fig. 7 (a), (b), and (c) it can be seen that the leukocyte mask is shown in white color whereas, the background of the blood sample image is black.

#### Noise removal in leukocyte mask

**Fig. 8:**
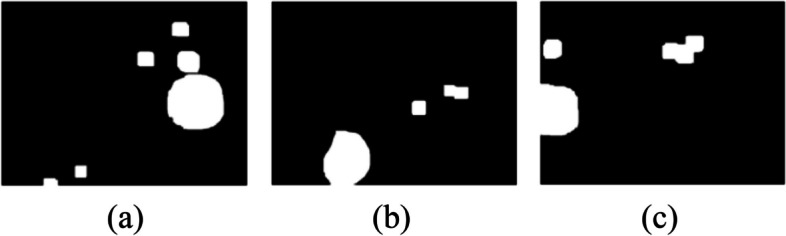
Noise removal in leukocyte mask

To remove the noise from the leukocyte mask morphological operations are used. From Fig. 7 shown in the last section, it can be seen that noise is present in leukocyte mask images. To get rid of the little scraps and make the masks rounder, opening morphological operation is used i.e. erosion followed by dilation. Figure 8 shows the removed noise images of leukocyte masks. The equations of dilation, erosion, and opening are given in Eqs. 4, 5, and 6 respectively. The equation of dilation operation is

4 $$P \oplus Q=\left\{R|\left[\left(Q{\wedge }_{z}\right)\cap P\right]\epsilon P\right\}$$

Here, $P$ is the image and $Q$ is the structuring element. $\left(Q\wedge \_z\right)$ it means to take a reflection of $Q$ about its origin and shift it by $R$. Therefore, dilation of $P$ with $Q$ is a set of all displacements, $R$ such that $\left(Q\wedge \_z\right)$ and $P$ overlap by at least one element. The equation of erosion operation is

5 $$P \circleddash Q=\left\{R|\left(Q\wedge \_z\right)\epsilon P\right\}$$

Here, the erosion of $P$ by $Q$ is a set of all points that $Q$, shifted by $R$ is a subset of $P$ that is $Q$ is entirely contained within $P$. Erosion reduces the number of pixels from the object boundary. The equation of opening operation is

6 $$P \circ Q=OPEN\left(P,Q\right)=\left(P \circleddash Q\right) \oplus Q$$

Morphological opening of an image is erosion followed by dilation.

#### Bounding boxes of the selected leukocytes

**Fig. 9:**
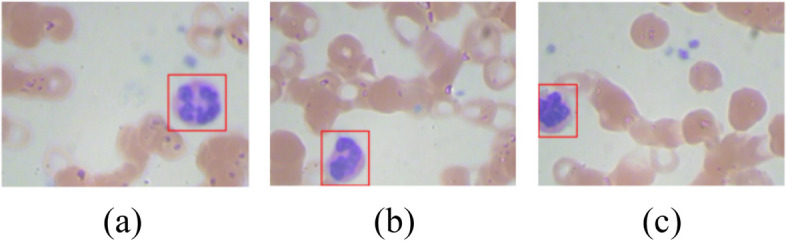
Creating bounding boxes for the selected leukocyte

In this step, the mask having the highest area in the image sample is marked as leukocyte with the bounding boxes and the outline color is made red as shown in Fig. 9.

#### Image cropping to get isolated leukocytes

**Fig. 10:**
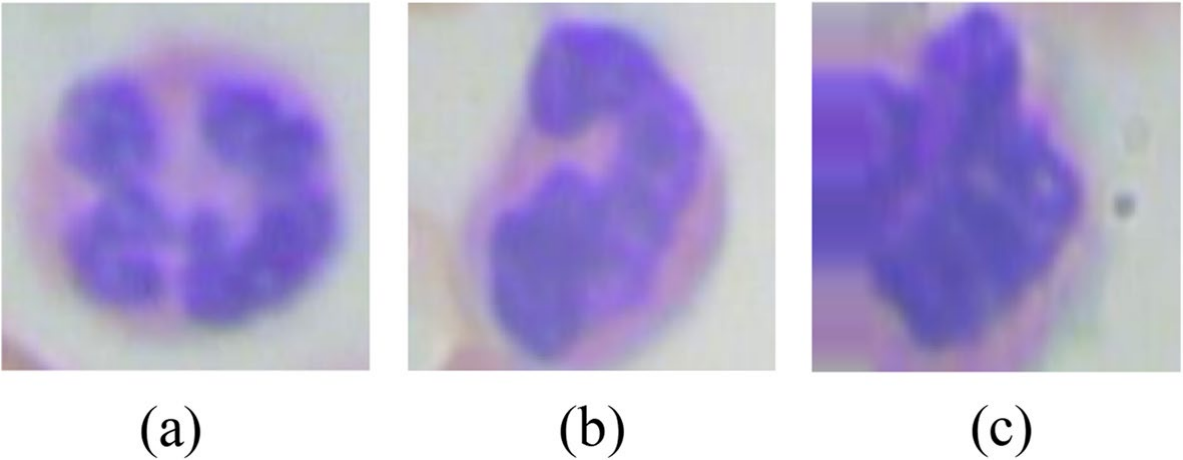
Isolated leukocytes

In this step, the selected leukocyte image is cropped to an image size of 128 * 128 as shown in Fig. 10.

### Data augmentation

Overfitting is a challenge for large neural networks in biological applications since only a relatively small number of datasets are available. The dataset is therefore expanded to achieve greater performance.

**Fig. 11:**
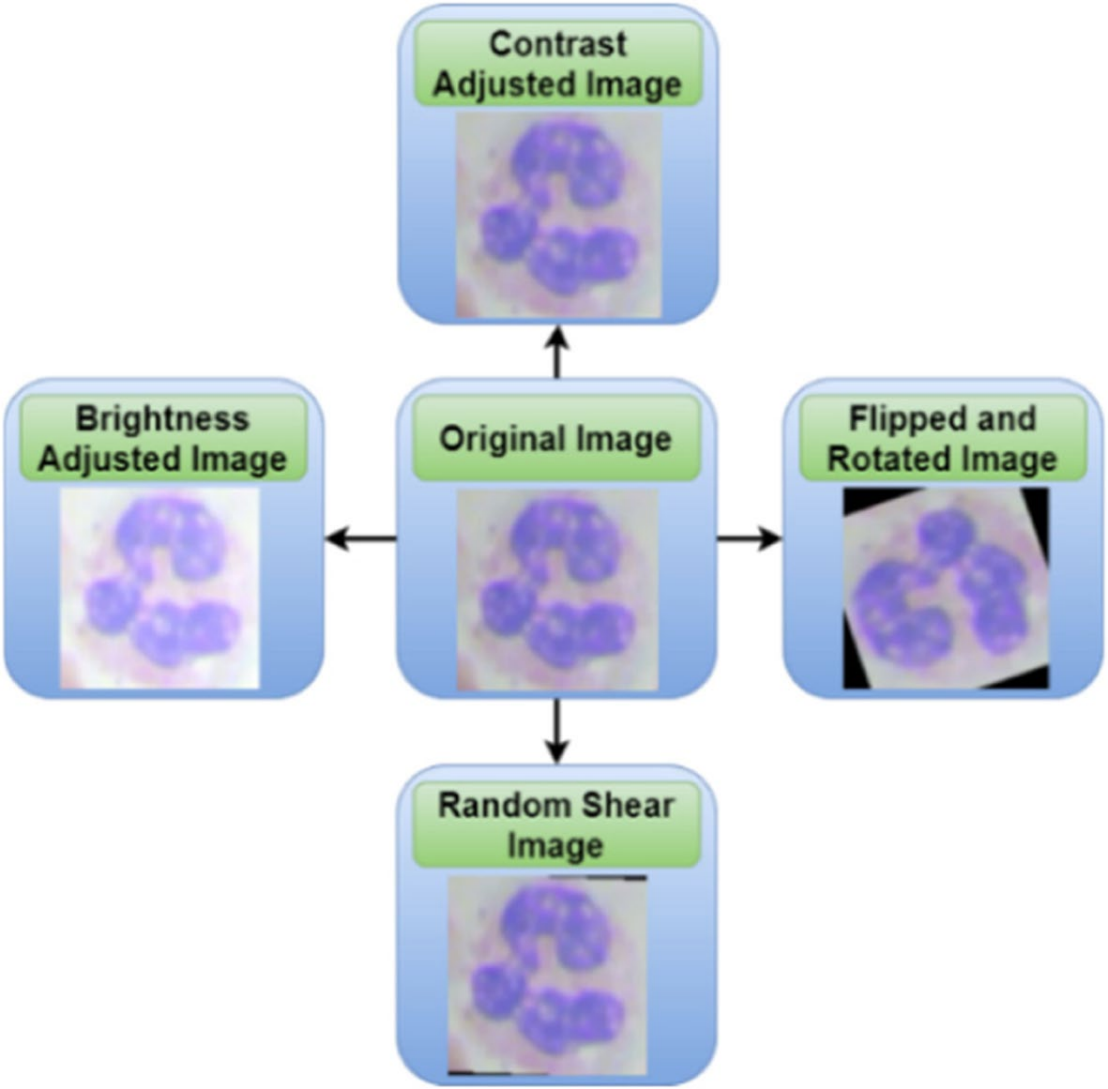
Data augmentation techniques

Various operations such as rotation, flipping, contrast, brightness, and random shear are applied to each image input during data augmentation as shown in Fig. 11. Thereby increasing the overall training image count is a good thing. Images are then divided into two sets: one for training and another for testing reasons. The CNN model is used to train the model.

### Deep convolution neural network architecture for segmented leukocyte

**Fig. 12:**
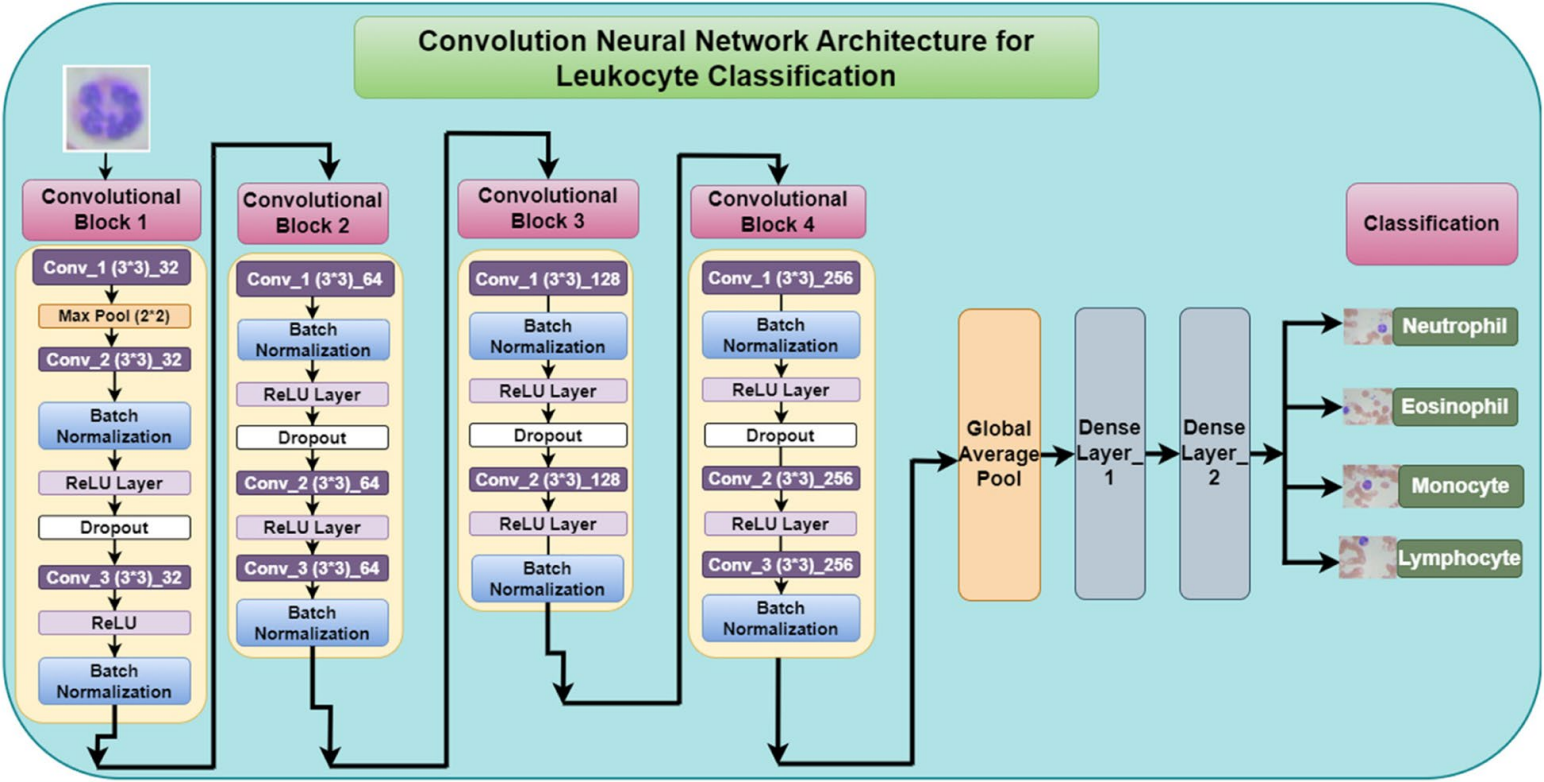
Convolutional Neural Network Architecture for leukocyte Classification

The convolutional neural network (CNN) architecture in the Fig. 12 is a four-block deep learning model designed to classify leukocytes in blood smear images. Convolutional layer applies filters (learned kernels) that slide across the input image, extracting features like edges and textures. The number of filters increases progressively through the blocks, allowing the network to study progressively complex features. ReLU activation layer introduces non-linearity into the network, enabling it to learn complex relationships between features. Batch normalization layer reduces internal covariate shift, stabilizing the training process and improving the model's generalizability.

Following these convolutional blocks, a global average pooling layer captures spatial information from the feature maps, producing a fixed-size output that's suitable for feeding into the final densely connected output layer. This final layer has four output neurons, corresponding to the four leukocyte classes i.e. neutrophil, eosinophil, lymphocyte, and monocyte.

The first convolutional block consists of 3 convolution layers with filter size of 3 * 3 and a total of 32 filters, 2 batch normalization layers, 1 dropout, and 1 max pool layer with 2 * 2 filter size with 2 ReLU layers.

The second convolutional block contains 3 convolution layers with filter size of 3 * 3 and a total of 64 filters, 2 batch normalization layers, 1 dropout, and 2 ReLU layers. The third convolutional block contains two convolution layers with filter size of 3 * 3 and 128 total filters, two batch normalization layers and two ReLU layers. The fourth convolutional block consists of three convolution layers, two batch normalization layers, two ReLU layers and one dropout layer. After the 4 convolutional blocks, the global average pooling layer is attached with two dense layers. In the last step, the blood sample image is classified into the four classes named as Neutrophil, Eosinophil, Monocyte and Lymphocyte. Overall, this CNN architecture employs a step-wise approach, progressively extracting higher-level features from the input image to ultimately achieve accurate leukocyte classification.

The rationale behind using a deep Convolutional Neural Network (CNN) for leukocyte classification lies in its ability to automatically learn hierarchical features from raw data. Deep CNN has more convolution layers to capture more complex features in the leukocyte images to learn deep abstract information of the data, potentially leading to better performance. CNNs are well-suited for this task because they can capture spatial hierarchies of features in images through their convolutional layers, which apply filters to detect patterns at different spatial scales. The proposed approach has total parameters as 134,853, with 134,085 as Trainable parameters and 768 as Non-trainable parameters.

## Results and discussion

This segment shows the results attained using the proposed deep CNN model integrated with image processing methods. To analyse the presentation of proposed model, it is simulated in three ways. In the first case, model is simulated in such a way that neither segmentation of leukocyte is performed nor augmentation is done. In the second case, both segmentation as well as augmentation are performed in the proposed model. Thereafter, the classification results of these two cases are compared to analyse the best case. The best case is further compared with the state-of-art models. The segmentation result analysis cannot be performed for the proposed model because the ground truth for segmentation mask is not provided for this dataset. Hence, the performance of model is analysed in terms of classification accuracy, not in terms of segmentation accuracy. The model has been analyzed based on train loss, train accuracy, validation loss, validation accuracy, precision, sensitivity, F1-score, and accuracy. The fine-tuning of the model is performed using diverse hyperparameters like Adam optimizer, batch size value 32, and epochs.

### Results of proposed model without segmentation and augmentation

**Fig. 13:**
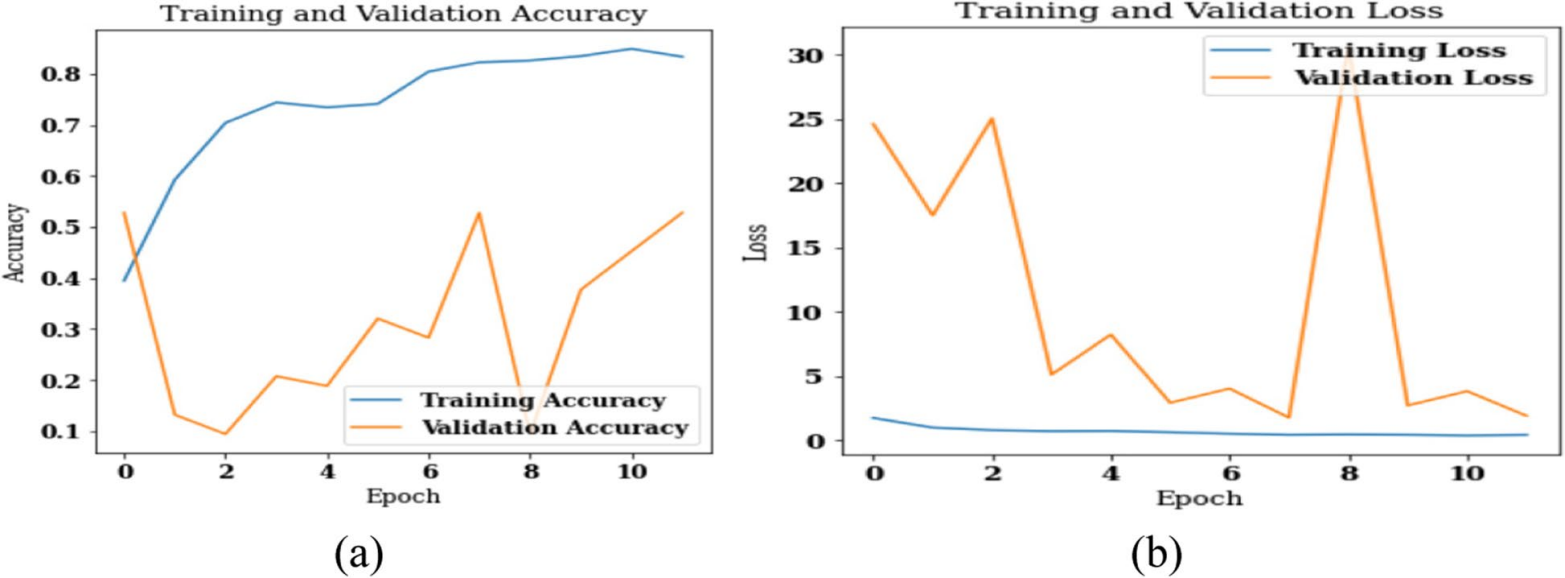
Results of proposed model without segmentation and augmentation (**a**) Training and Validation Accuracy, (**b**) Training and Validation Loss

Here, the proposed model is applied directly on the original images without data pre-processing techniques. The leukocyte region is not segmented and cropped from the original images. Moreover, no data augmentation technique is applied on the original or cropped images in this case. An analysis is performed based on confusion matrix parameters, Cohen’s Kappa score, training, and validation accuracy and loss curves. the model is simulated using the 32 batch size value and a total of 12 epochs. Figure 13 shows the training and validation loss and accuracy curves. Figure 13 (a) shows the accuracy curves and it can be analyzed from the figure that the value of training accuracy is approximately 80% and the value of validation accuracy is approximately 55%. Similarly, Fig. 13 (b) shows the loss curves and it is observed that the value of validation loss is between 0-2.

**Fig. 14:**
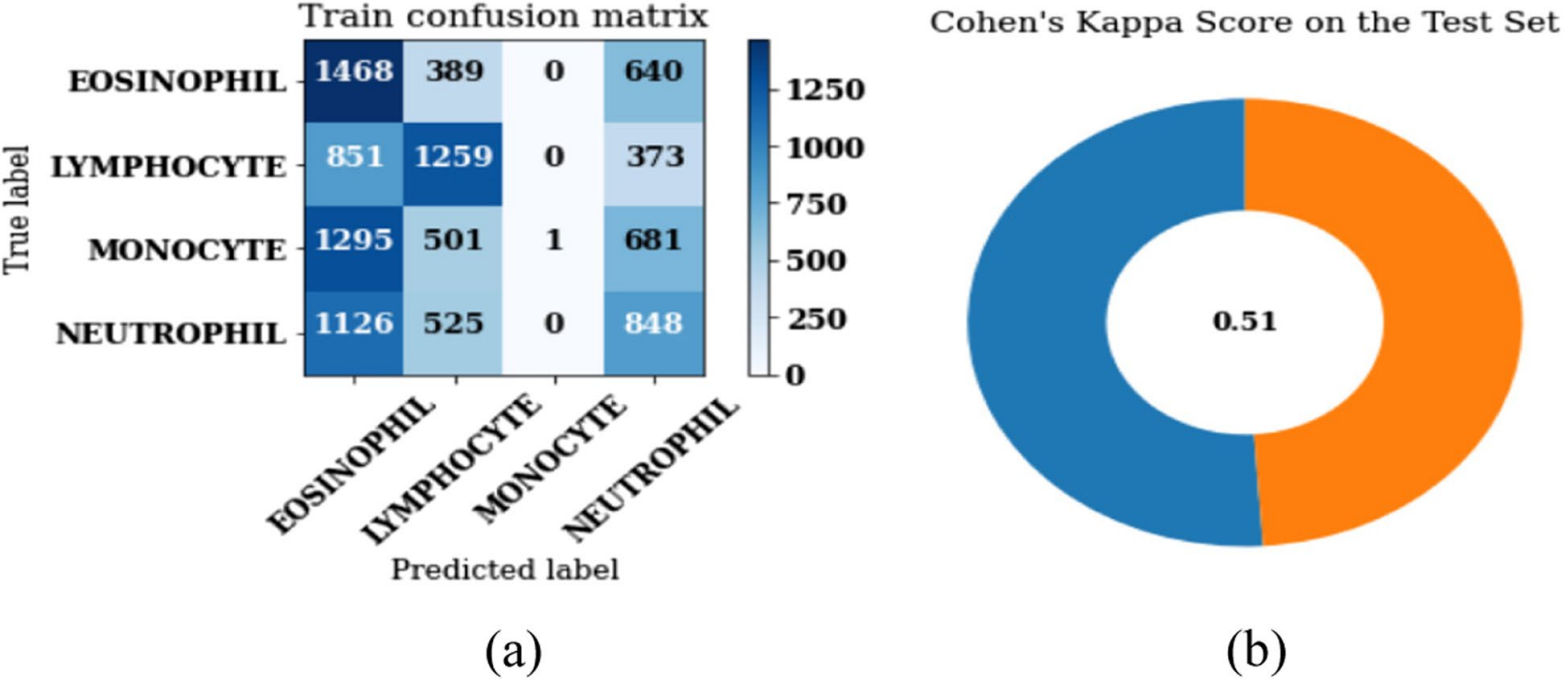
Results of proposed model without segmentation and augmentation (**a**) Confusion matrix, (**b**) Cohen’s Kappa Score

Figure 14 (a) shows the confusion matrix of the proposed model on batch size 32 and Adam optimizer. In a matrix, the number of images classed by a given model can be determined by the diagonal values of the matrices. Figure 14 (b) shows the Cohen’s Kappa score on the test set. Cohen's Kappa statistic is utilized to determine how well two raters or judges agree on the classification of an item into two distinct groups. The Cohen’s Kappa can be calculated as:

7 $$K=\left(PO-PE\right)/\left(1-PE\right)$$

Here, $PO$ is the Relative observed agreement among raters and $PE$ is the Hypothetical probability of chance agreement. The Cohen's kappa score is a statistical measure used to assess the agreement between two raters or classifiers. It considers the agreement that would be expected by chance and then normalizes the observed agreement by this value. This normalization accounts for the possibility of random agreement, providing a more robust measure of agreement. The kappa score ranges from -1 to 1, where 1 indicates perfect agreement, 0 indicates agreement equivalent to chance, and values less than 0 indicate agreement worse than chance. It is commonly used in the evaluation of classification models, particularly in cases where the classes are imbalanced. The value of Cohen Kappa score is 0.51 on the test set.

From the confusion matrix, various considerations such as sensitivity, precision, F1-score and accuracy are calculated and shown in Table 1. The overall accuracy value of the model without segmentation and augmentation obtained is 79%. In case of precision, the model is performing best for lymphocyte type with the value as 83%, whereas in case of sensitivity and F1-score, the model is showing best value for neutrophil as 90% and 83% respectively.

**Table 1:** Performance parameters of the proposed model without segmentation and augmentation

**Leukocyte Type**	**Precision (%)**	**Sensitivity (%)**	**F1-Score**	**Accuracy (%)**
Neutrophil	77	90	83	79
Eosinophil	65	50	56	
Monocyte	67	40	50	
Lymphocyte	83	62	71	

### Results of the proposed model with segmentation and augmentation

Here, the proposed model is applied on the segmented images. The leukocyte region is segmented and cropped from the original images. Also, data augmentation technique is applied on the segmented or cropped images in this case.

**Fig. 15:**
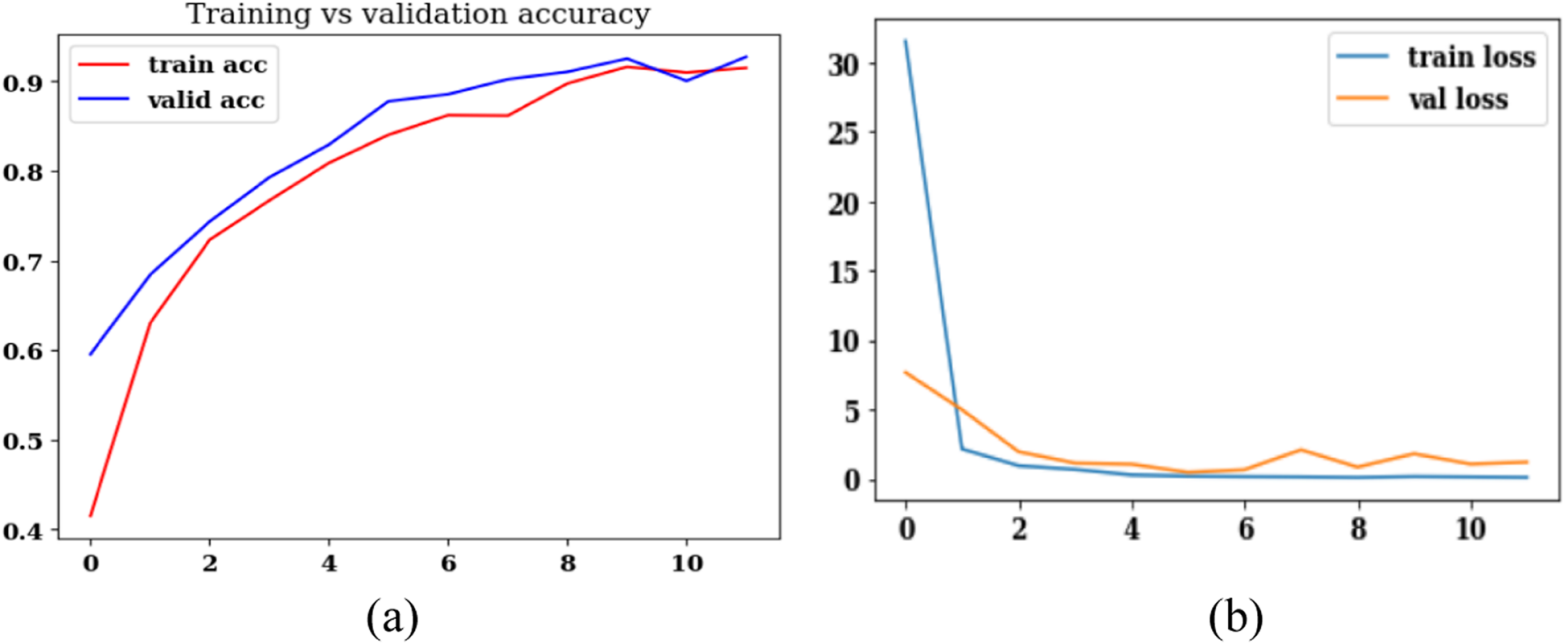
Results of the proposed model with segmentation and augmentation (**a**) Training and Validation Accuracy, (**b**) Training and Validation Loss

**Fig. 16:**
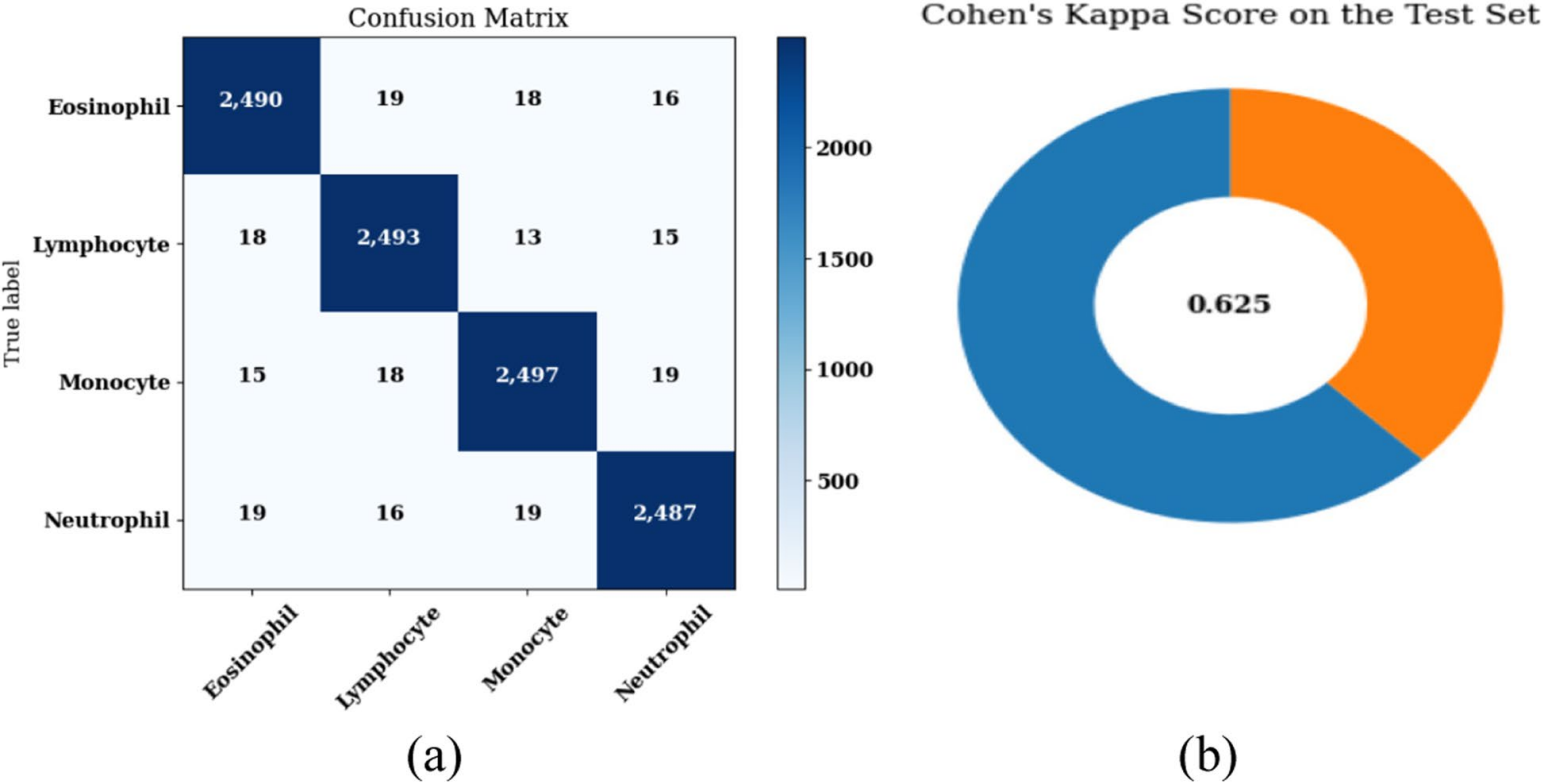
Results of the proposed model with segmentation and augmentation (**a**) Confusion matrix, (**b**) Cohen’s Kappa Score

An analysis is performed based on confusion matrix parameters, training, and validation accuracy and loss curves, Cohen’s Kappa score. Figure 15 displays the validation and training loss, accuracy curves for a total of 12 epochs. Figure 15 (a) shows the accuracy curves and it can be analyzed from the figure that the value of training accuracy is near 92%. Similarly, Fig. 15 (b) shows the loss curves and the value of the loss is constant after the 2^nd^ epoch. The loss value is declining with the increase in the epoch value. Figure 16 (a) shows the confusion matrix of the proposed model on batch size 32 and Adam optimizer. Figure 16 (b) shows the Cohen’s Kappa score on the test set. The value of Cohen’s Kappa score is 0.625 that is ranging between 0-1.

From the confusion matrix, various considerations such as sensitivity, precision, F1-score and accuracy are considered and shown in Table 2. The overall accuracy value of the model with segmentation and augmentation obtained is 91.18%.

**Table 2:** Performance parameters of the proposed model with segmentation and augmentation

**Leukocyte Type**	**Precision (%)**	**Sensitivity (%)**	**F1-Score**	**Specificity**	**Accuracy (%)**
Neutrophil	97.95	97.91	97.93	99.31	97.98
Eosinophil	97.91	98.18	98.05	99.30	
Monocyte	98.03	97.95	97.99	99.34	
Lymphocyte	98.02	97.87	97.95	99.33	

### Ablation analysis for the proposed model

The performance comparison of proposed model is simulated in two ways. In the first way, the proposed model is applied directly on the original images without data pre-processing techniques. The leukocyte region is not segmented and cropped from the original images. Moreover, no data augmentation technique is applied on the original or cropped images in this case. In this case the proposed model is simulated on the various performance factors such as accuracy, precision, sensitivity and F1 score. In the second way, the proposed model is applied on the segmented images. The leukocyte region is segmented and cropped from the original images. Also, data augmentation technique is applied on the segmented or cropped images in this case.

**Fig. 17:**
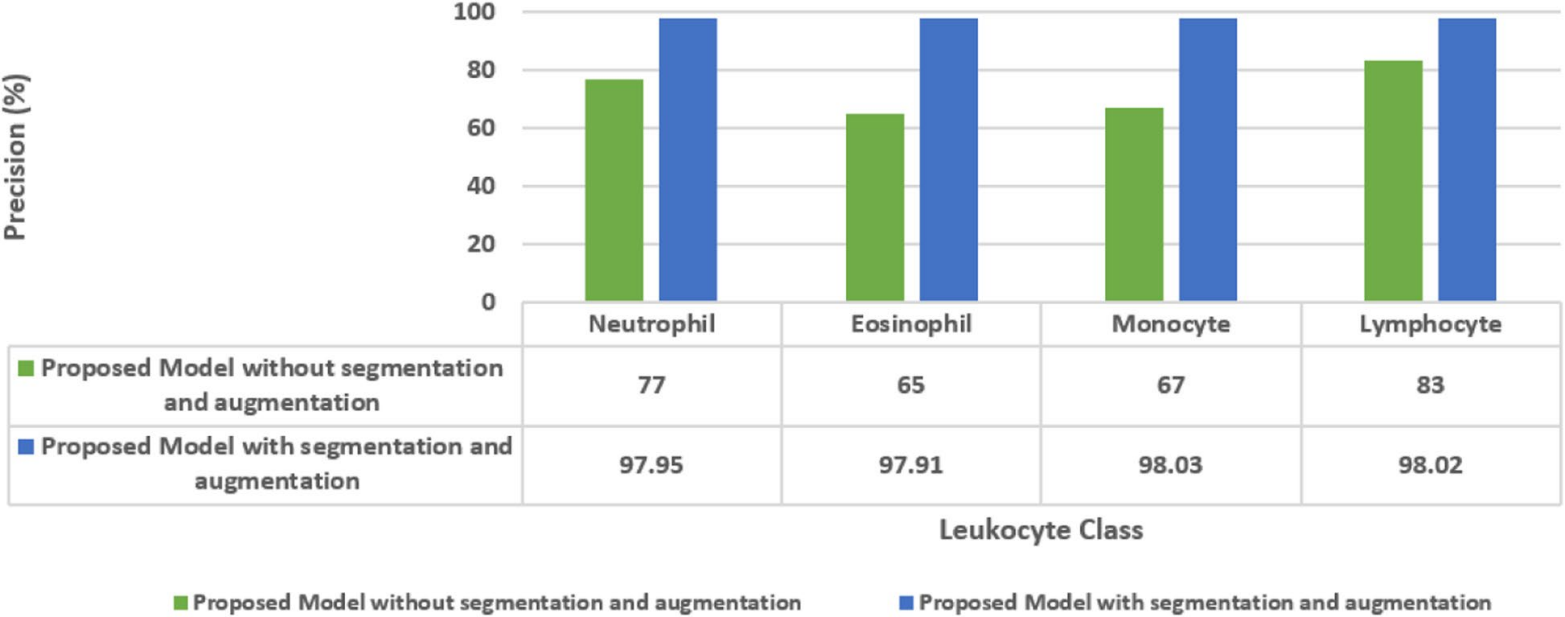
Ablation analysis for the proposed model in terms of precision

Figure 17 shows the ablation analysis of the proposed approach. Without segmentation and augmentation, the model achieved moderate precision ranging from 65% to 83% for different leukocyte types. However, with the inclusion of segmentation and augmentation, there was a substantial improvement in precision, with values ranging from 97.91% to 98.03%. This significant increase demonstrates the effectiveness of segmentation and augmentation in enhancing the model's performance, particularly in accurately classifying neutrophils, eosinophils, monocytes, and lymphocytes in human blood images.

**Fig. 18:**
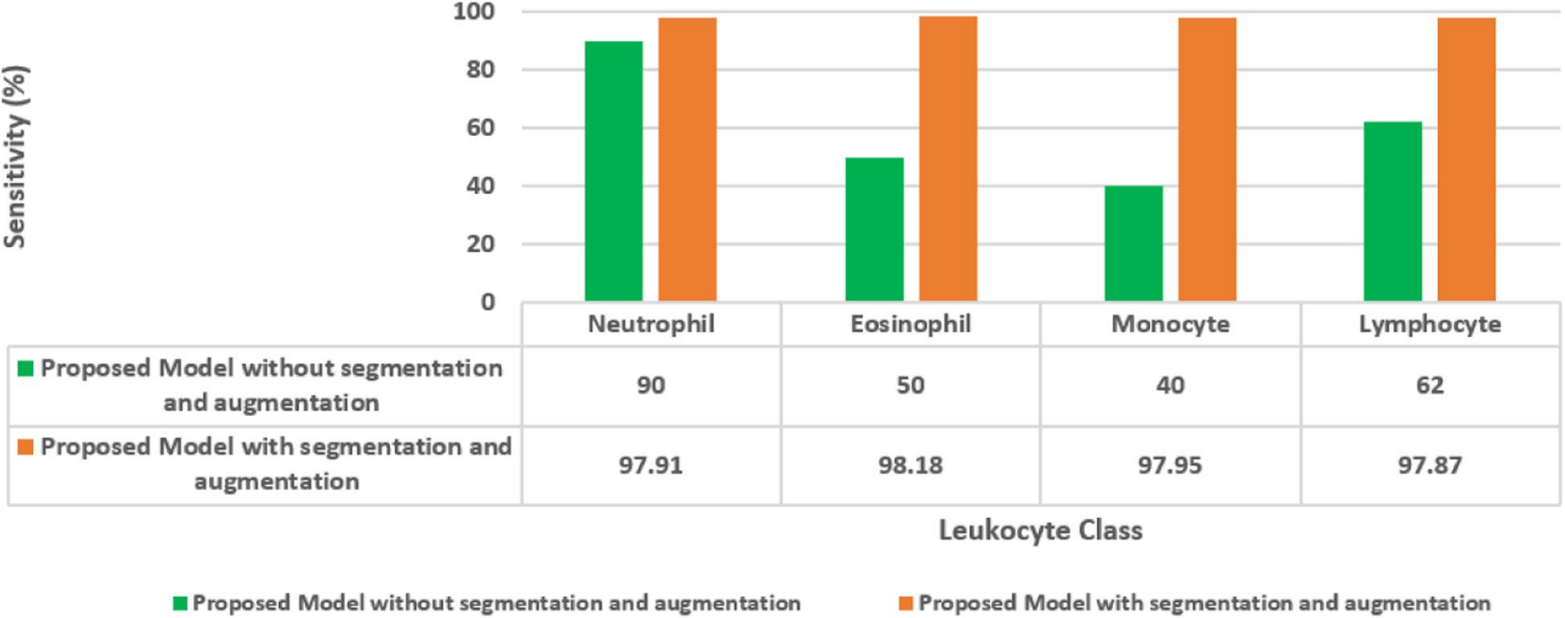
Ablation analysis for the proposed model in terms of sensitivity

The Fig. 18 showcases the sensitivity values of a proposed model for leukocyte classification, comparing outcomes without segmentation and augmentation to those with these techniques. Without segmentation and augmentation, the model exhibited varying sensitivities for different leukocyte types, ranging from 40% to 90%. However, with segmentation and augmentation, there was a notable improvement in sensitivity across all classes, with values ranging from 97.87% to 98.18%. This enhancement highlights the effectiveness of segmentation and augmentation in improving the model's ability to correctly identify neutrophils, eosinophils, monocytes, and lymphocytes in human blood images, particularly evident in the substantial increase in sensitivity for eosinophils and monocytes.

**Fig. 19:**
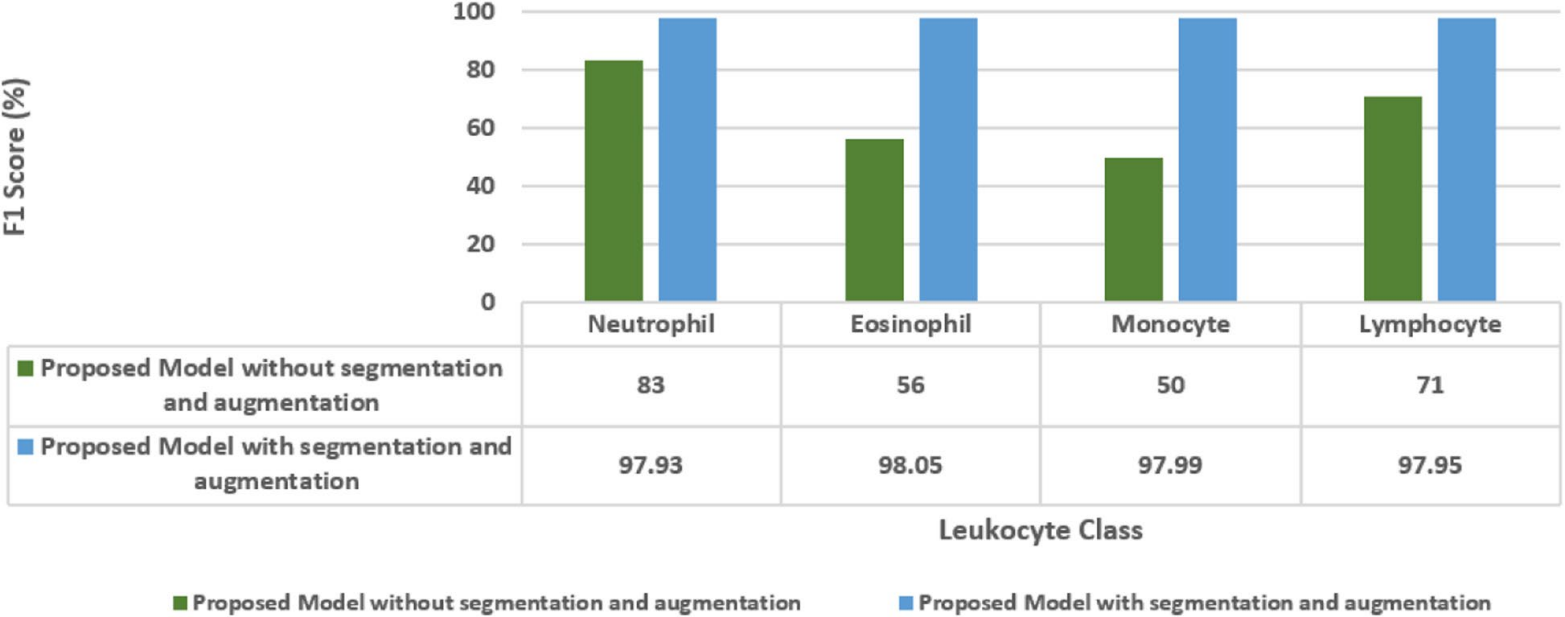
Ablation analysis for the proposed model in terms of F1-score

The Fig. 19 displays the F1-scores of a proposed model for leukocyte classification, comparing results without segmentation and augmentation to those with these techniques. Without segmentation and augmentation, the model achieved moderate F1-scores ranging from 50% to 83% for different leukocyte types. However, with segmentation and augmentation, there was a significant improvement in F1-scores across all classes, with values ranging from 97.93% to 98.05%. This substantial increase demonstrates the effectiveness of segmentation and augmentation in enhancing the model's ability to balance precision and recall, particularly evident in the remarkable improvement in F1-scores for eosinophils, monocytes, and lymphocytes.

### Comparison with state-of-art

The evaluation of the proposed model with state-of-art methods is shown in Table 3. It illustrates that the proposed framework has been successfully utilizing segmentation and classification both, whereas the current state-of-the-art techniques has worked on classification only.

**Table 3:** Comparison of the proposed model with existing state-of-art models

**Reference**	**Technique Used**	**Segmentation**	**Classification**	**Performance Parameters**			
				**Accuracy (%)**	**Precision (%)**	**Sensitivity (%)**	**F1-Score**
Liang et al. [21]	CNN-RNN	**×**	✓	95.42	91.75	96.91	89.0
Pang et al. [22]	Fusion of Shallow and Deep Feature Maps	**×**	✓	94.33	91.25	96.00	89.0
Yu et al. [23]	Deep Neural Network	**×**	✓	90.51	81.5	92.39	81.0
Banik et al. [24]	Feature Map Fusion of Two Convolution Layers	**×**	✓	96.01	97	99.03	92.0
Proposed Model	Deep Feature Map Extraction of Segmented Leukocyte	✓	✓	97.98	97.97	97	97.0

## Conclusion and future work

Leukocyte (WBCs) are in charge of the immune system in human blood that protects the human body from diseases, parasites and bacteria. The manual count of WBCs and its classification into its four types such as lymphocytes, monocytes, eosinophils, and neutrophils is common practice among hematopathologists to identify leukemia. But this is a time consuming and laborious task that necessitate the assistance of medical professionals. Moreover, due to the intensity variance and imaging conditions of blood images, WBC segmentation is challenging task. Thus, there is necessity for a computer-aided system that can segment and classify leukocytes automatically to analyze human blood. In this work, a deep learning based model has been proposed for segmentation and classification of leukocytes into its four different types. The proposed model is estimated independently on each of the four types of leukocyte images, and it shows good precision, sensitivity, accuracy, and F1 score. Overall, this method achieves a higher classification accuracy of 97.98% than any other state-of-art techniques.

The proposed deep learning model for leukocyte segmentation and classification has significant practical implications for medical diagnostics and patient care. By automating the laborious and time-consuming task of manual leukocyte counting and classification, the model can improve the efficiency of hematopathologists and reduce diagnostic errors. This could lead to faster and more accurate diagnoses of leukemia and other blood-related disorders, ultimately improving patient outcomes.

In future work, the proposed model's performance can be enhanced by incorporating more advanced deep learning architectures and algorithms, such as attention mechanisms or graph convolutional networks, to improve feature extraction and classification accuracy. Furthermore, the model's capabilities can be extended to handle additional leukocyte subtypes or abnormalities, thereby increasing its applicability in a broader range of hematological analyses. Finally, we intend to validate the proposed model on a larger and more diverse dataset to ensure its robustness and generalizability.

## Data Availability

The images are collected from a publicly available dataset from Kaggle. The datasets used and/or analyzed in the current study are available from the first author upon reasonable request.

## Abbreviations

CNN: Convolutional Neural Network
ReLU: Rectified Linear Unit
LDA: Linear Discriminant Analysis
NE: Neutrophil
EO: Eosinophil
LM: Lymphocyte
MN: Monocyte

## References

[1] Roy RM, Ameer PM (2021). Segmentation of leukocyte by semantic segmentation model: a deep learning approach. Biomed Signal Process Control.

[2] Anto Bennet M, Diana G, Pooja U, Ramya N. Texture metric driven acute lymphoid leukemia classification using artificial neural network. Int J Recent Technol Eng (IJRTE). 2019; 7(6S3):152–156.

[3] Neoh SC, Srisukkham W, Zhang L, Todryk S, Greystoke B, Lim CP, Hossain MA (2015). Aslam N an intelligent decision support system for leukaemia diagnosis using microscopic blood images. Sci Rep.

[4] ElDahshan KA, Youssef MI, Masameer EH, Mustafa MA (2015). An efficient implementation of acute lymphoblastic leukemia images segmentation on the FPGA. Adv Image Vid Process.

[5] Bhattacharya T, Soares GABE, Chopra H, Rahman MM, Hasan Z, Swain SS, Cavalu S (2022). Applications of phyto-nanotechnology for the treatment of neurodegenerative disorders. Materials.

[6] Sharma B, Koundal D (2018). Cattle health monitoring system using wireless sensor network: a survey from innovation perspective. IET Wireless Sensor Syst.

[7] Jabeen K, Khan MA, Hameed MA, Turki-Hadj M and Masood A. A Novel Fusion Framework of Deep Bottleneck Residual Convolutional Neural Network for Breast Cancer Classification from Mammogram Images. Front Oncol. 2024;14. 10.3389/fonc.2024.1347856 PMC10917916 38454931

[8] Ullah MS, Khan MA, Masood A, Mzoughi O, Saidani O and Alturki N. Brain tumor classification from MRI scans: a framework of hybrid deep learning model with Bayesian optimization and quantum theory-based marine predator algorithm. Front Oncol. 2024;14:1–21. 10.3389/fonc.2024.1335740 PMC10882068 38390266

[9] Rauf F, Khan MA, Bashir AK, Jabeen K, Hamza A, Alzahrani AI, Alalwan N. and Masood A. Automated deep bottleneck residual 82-layered architecture with Bayesian optimization for the classification of brain and common maternal fetal ultrasound planes. Front Med. 2023;10:1–14. 10.3389/fmed.2023.1330218 PMC10769562 38188327

[10] Rezatofighi SH, Soltanian-Zadeh H (2011). Automatic recognition of five types of white blood cells in peripheral blood. Comput Med Imaging Graph.

[11] Cao H, Liu H, Song E (2018). A novel algorithm for segmentation of leukocytes in peripheral blood. Biomed Signal Process Control.

[12] Saidi M, El Amine Bechar M, Settouti N, Chikh MA. Application of pixel selection in pixel-based classification for automatic white blood cell segmentation. Proc Mediterr Conference Pattern Recogn Artif Intell. 2016:31–8.

[13] Zheng X, Wang Y, Wang G, Liu J (2018). Fast and robust segmentation of white blood cell images by self-supervised learning. Micron.

[14] Daqqa KASA, Maghari AYA, Sarraj WFMA (2017). Prediction and diagnosis of leukemia using classification algorithms. 2017 8^th^ international conference on information technology (ICIT).

[15] Tatdow Pansombut, WikaisuksakulSiripen, KhongkraphanKittiya, Phon-onAniruth. Convolutional neural networks for recognition of lymphoblast cell images. Comput Intell Neurosci. 2019:1–12. 10.1155/2019/7519603 PMC6589284 31281337

[16] Aliyu HA, Sudirman R, Abdul Razak MA, Abd Wahab MA (2018). Red blood cell classification: deep learning architecture versus support vector machine. 2018 2^nd^ international conference on biosignal analysis, processing and systems (ICBAPS).

[17] Mohammad Zhana F, Abdulla AA. Thresholding-based white blood cells segmentation from microscopic blood images. UHD J Sci Technol. 2020;4(1):9–17.

[18] Shahin AI, Guo Y, Amin KM, Sharawi AA (2019). White blood cells identification system based on convolutional deep neural learning networks. Comput Methods Progr Biomed.

[19] Mishra S, Majhi B (2019). PK Sa, Texture feature based classification on microscopic blood smear for acute lymphoblastic leukemia detection. Biomed Signal Process Control.

[20] Mooney “Kaggle Dataset https://www.kaggle.com/datasets/paultimothymooney/blood-cells”.

[21] Liang G, Hong H, Xie W, Zheng L (2018). Combining convolutional neural network with recursive neural network for blood cell image classification. IEEE Access.

[22] Pang S, Du A, Orgun MA, Yu Z (2019). A novel fused convolutional neural network for biomedical image classification. Med Biol Eng Comput.

[23] Yu W, Chang J, Yang C, Zhang L, Shen H, Xia Y, Sha J (2017). Automatic classification of leukocytes using deep neural network. IEEE 12^th^ international conference on ASIC (ASICON).

[24] Banik PP, Saha R, Kim K-D (2019). Fused convolutional neural network for white blood cell image classification. International conference on artificial ıntelligence in information and communication.

